# Natural Autoimmunity to the Thyroid Hormone Monocarboxylate Transporters MCT8 and MCT10

**DOI:** 10.3390/biomedicines9050496

**Published:** 2021-04-30

**Authors:** Theresa Porst, Jörg Johannes, Hans Gluschke, Richard Köhler, Sebastian Mehl, Peter Kühnen, Kostja Renko, Waldemar B. Minich, Susanna Wiegand, Lutz Schomburg

**Affiliations:** 1 Institute for Experimental Endocrinology, Charité-Universitätsmedizin Berlin, Corporate Member of Freie Universität Berlin and Humboldt Universität zu Berlin, D-10115 Berlin, Germany; theresaporst@gmx.de (T.P.); jjohannes@web.de (J.J.); hans.gluschke@charite.de (H.G.); richard.koehler@charite.de (R.K.); sebastian.mehl@mac.com (S.M.); kostja.renko@charite.de (K.R.); waldemar.minich@charite.de (W.B.M.); 2 Department of Paediatric Endocrinology and Diabetology, Charité-Universitätsmedizin Berlin, Corporate Member of Freie Universität Berlin and Humboldt Universität zu Berlin, D-13353 Berlin, Germany; peter.kuehnen@charite.de (P.K.); susanna.wiegand@charite.de (S.W.)

**Keywords:** thyroid hormone, transport, thyroid axis, autoimmunity

## Abstract

The monocarboxylate transporters 8 (MCT8) and 10 (MCT10) are important for thyroid hormone (TH) uptake and signaling. Reduced TH activity is associated with impaired development, weight gain and discomfort. We hypothesized that autoantibodies (aAb) to MCT8 or MCT10 are prevalent in thyroid disease and obesity. Analytical tests for MCT8-aAb and MCT10-aAb were developed and characterized with commercial antiserum. Serum samples from healthy controls, thyroid patients and young overweight subjects were analyzed, and prevalence of the aAb was compared. MCT8-aAb were additionally tested for biological effects on thyroid hormone uptake in cell culture. Positive MCT8-aAb and MCT10-aAb were detected in all three clinical cohorts analyzed. MCT8-aAb were most prevalent in thyroid patients (11.9%) as compared to healthy controls (3.8%) and overweight adolescents (4.2%). MCT8-aAb positive serum reduced T4 uptake in cell culture in comparison to MCT8-aAb negative control serum. Prevalence of MCT10-aAb was highest in the group of thyroid patients as compared to healthy subjects or overweight adolescents (9.0% versus 4.5% and 6.3%, respectively). We conclude that MCT8 and MCT10 represent autoantigens in humans, and that MCT8-aAb may interfere with regular TH uptake and signaling. The increased prevalence of MCT8-aAb and MCT10-aAb in thyroid disease suggests that their presence may be of pathophysiological relevance. This hypothesis deserves an analysis in large prospective studies.

## 1. Introduction

The majority of thyroid hormones (TH) circulating in blood are not free, but bound to TH binding proteins due to their hydrophobic nature, namely to albumin, thyroxine binding globulin, and transthyretin, respectively. In order to exert TH signaling via the nuclear TH receptors, TH need to pass the plasma membrane of target cells [1]. However, TH are charged amino acid derivatives and unable to diffuse across the hydrophobic membrane segments. Transmembrane proteins enabling passive TH passage or capable of actively transporting TH are therefore essentially needed [2]. There is a large number of potential TH transporters differing in structure, expression pattern, transport preferences and regulation of biosynthesis, activity and trafficking [3]. The family of monocarboxylate transporters (MCT) are of specific interest as MCT8 (solute carrier family 16 member 2, SCL16A2) was the first TH transporter identified to be causally involved in an inherited human disease, i.e., causative for the Allan–Herndon–Dudley syndrome (AHDS) [4,5]. The gene encoding MCT8 (*SLC16A2*) is located on the X-chromosome, and transport-impeding mutations in *SLC16A2* are interfering with regular TH signaling, thereby disturbing muscular, neuronal and intellectual development. The affected children display severe congenital hypotonia and may develop spasticity and generalized muscle weakness [6]. The disease often presents with a debilitating phenotype, apparently caused by severe cerebral hypothyroidism in combination with peripheral thyrotoxicosis [7]. Different therapeutic routes are discussed [8], and first clinical trials are conducted in young and adult patients affected by AHDS [9]. In case of prenatal diagnosis, therapy is attempted by treating the fetus via the pregnant mother [10]. MCT10 (SCL16A10) constitutes a second TH transporter of high similarity to MCT8 both in sequence, structure and transport characteristics [11]. Genetic defects in *SCL16A10* have not been identified, yet.

Besides mutations and polymorphisms in the genes encoding central components of the TH axis, circulating autoantibodies (aAb) are also capable of interfering with regular TH status and feedback control. The G-protein coupled thyrotropin (TSH) receptor (TSHR) of the thyroid gland is probably the most illuminating example of a central component of the TH axis that can be affected by the immune system, as neutral, blocking and stimulating aAb to the TSHR have been identified and characterized [12,13]. The detection and characterization of TSHR-aAb is the leading criterion in the diagnosis of Graves’ disease [14,15]. 

With the characterization of MCT8 as an essential plasma membrane transporter for peripheral and central TH uptake that is directly exposed to the circulation, we decided to test whether MCT8 may constitute an autoantigen in human subjects. To this end, we established novel quantitative aAb assays to human MCT8 and in parallel to the highly related MCT10 molecule, and assessed the prevalence of MCT8-aAb and MCT10-aAb in three groups of subjects, i.e., healthy adults, overweight adolescents and thyroid patients. The inclusion of a group of obese subjects was of particular relevance, as obesity increases the risk for autoimmunity due to the interference of fat-derived adipokines with the immune system [16,17,18,19], and the potential consequence of autoimmune thyroid disease and a suppressed TH status causing weight gain and diabetes risk [20,21,22]. The rationale for choosing these groups of subjects lies in the hypothesis that inhibitory aAb to MCT8 or MCT10 would modify thyroid gland function, TH status and energy homeostasis. Indeed, positive subjects for MCT8-aAb and MCT-10-aAb were identified and a particularly elevated prevalence of MCT8-aAb was observed in thyroid disease.

## 2. Materials and Methods

### 2.1. Human Samples

For the assessment of the prevalence of MCT8-aAb and MCT10-aAb in the general population, serum samples from adult subjects (*n* = 400, 50% females, self-reported health status as healthy) were purchased from a commercial supplier (in.vent Diagnostica GmbH, Hennigsdorf, Germany). Ethical clearance and written informed consent had been granted to the commercial supplier who provided the samples after pseudonymization. A potential relevance of MCT8-aAb and MCT10-aAb for metabolism and weight control was tested in a cohort of obese adolescents (*n* = 142) participating in the “MAINTAIN” intervention trial for body weight reduction [23]. Serum samples had been collected at the obesity outpatient clinic of Paediatric Endocrinology and Diabetology, Charité-Universitätsmedizin Berlin, Germany. Informed consent of the subjects and/or one or both parent(s) was obtained prior to study entry, and the study had been registered at Clinical Trials (NCT00850629). 

A potential relation of MCT8-aAb and MCT10-aAb to thyroid disease was tested in a cross-sectional study of adult thyroid patients visiting an outpatient clinic (*n* = 318). The study was conducted in Berlin, Germany, and patients were enrolled consecutively independent of thyroid disease type and ongoing therapy. All participants provided written informed consent before enrolment [24]. Both clinical studies had been approved by the Ethical committee of Charité-Universitätsmedizin, Berlin, in 2009 and 2017, respectively (#EA2/015/09 and #EA2/173/17). The studies were conducted in accordance with the guidelines in the Declaration of Helsinki. All samples were analyzed by personnel blinded to the clinical characteristics, and in a research lab remote from the clinical sites.

### 2.2. Commercial Antibodies

In order to test assay performance, suitability for clinical analyses, and to gain some insight into the detection limit of the newly generated assays, one commercial antiserum to human MCT8 and one to human MCT10, respectively, were purchased (novus biologicals, Europe Office Bio-Techne GmbH, Wiesbaden, Germany, cat. no. NBP2-57308, lot #A117467, and Sigma-Aldrich Chemie GmbH, Taufkirchen, Germany, prestige antibodies, cat. no. HPA016860, lot #B106546). The commercial antiserum samples were diluted in human serum as the preferred analytical matrix.

### 2.3. Construction of MCT8 and MCT10 Luciferase Fusion Proteins for aAb Detection

The open reading frames encoding human MCT8 and human MCT10, respectively, were amplified by PCR and fused in frame to a firefly luciferase gene (*Luc*), essentially as described [25,26,27]. The resulting composite reading frame was inserted into the expression vector pIRESneo, generating the expression vectors pIRESneo-MCT8-Luc and pIRESneo-MCT10-Luc, respectively. The sequences were verified by DNA sequencing using a commercial service supplier (LGC Genomics GmbH, Berlin, Germany). HEK293 cells were transfected and stable cell clones expressing high levels of recombinant MCT8-Luc and MCT10-Luc fusion protein, respectively, were established by applying selection pressure via the antibiotic geneticin (G418). Stably transfected cells were expanded in DMEM supplemented with 10% FBS and used for the production of sufficient amounts of the fusion proteins needed for the analyses. Briefly, confluent cells were grown in 75 mm^2^ dishes, harvested into PBS and lysed in resuspension buffer (20 mM HEPES-NaOH, pH 7.5, 100 mM NaCl, 1% Triton X-100, and 10% glycerol). The suspension was cleared by centrifugation (2000× *g*, 5 min, 4 °C), the supernatant was collected, stored at −80 °C in aliquots, and thawed when needed for the measurements. Precipitation assays were established with the transporter fusion proteins, essentially as described previously [25,26,27].

### 2.4. Quantification of MCT8 and MCT10 Autoantibodies 

Aliquots of the protein preparations (40 µL per reaction) were incubated with 10 µL of serum sample overnight at 4 °C. Immune complexes formed between the fusion proteins and endogenous immunoglobulins were then incubated with a protein A sepharose preparation (POROS-A, 10% (vol/vol), ASKA Biotech GmbH, Berlin) at room temperature for 1.5 h. Complexes were precipitated by centrifugation (500× *g*, 5 min, 4 °C), and pellets were washed three times with washing buffer (50 mM Tris-HCl, pH 7.5, 100 mM NaCl, 0.5% Triton X-100). Thereafter, luciferase activities were measured in a luminometer (Mitras, Berthold Technologies GmbH, Bad Wildbad, Germany), and results were recorded as relative light units (RLU). Binding indices (BI) were calculated as the ratio of the sample-specific signal observed in relation to the average of the bottom 50% of all signals, which was set at 1.0. Decision on positivity of a given signal was based on a mathematical outlier criterion using the full set of signals from the cohort of samples. To this end, the 75th percentile (P75) was determined, and 1.5-times the interquartile range (IQR) was added, yielding P75 + 1.5 × IQR as threshold. This type of analysis was conducted for each of the three cohorts separately, as each cohort was analyzed at a different time and showed slightly different levels of background, potentially due to the pre-analytical histories of the samples tested.

### 2.5. Characterization of MCT8-aAb by Immunoprecipitation and Thyroid Hormone Uptake

Hemagglutinin (HA)-tagged MCT8 (MCT8-HA) expressing Madin-Darby Canine Kidney (MDCK-1) cells had been generated before [28], and protein extract was prepared as described above. MCT8-HA containing cell extract (100 µL per reaction) was incubated with 50 µL of serum and 15 µL of protease inhibitor cocktail (cOmplete, Roche Diagnostics, Mannheim, Germany) overnight at 4 °C under constant agitation. The next day, samples were incubated with 100 µL of protein A slurry for 1.5 h at room temperature, and immune complexes formed were pelleted by centrifugation at (500× *g*). Precipitated material was washed six times with PBS and resuspended in DTT-containing sample buffer. The complexes were dissolved by incubation at 90 °C for 2 min. Samples were centrifuged at 10,000× *g* for 15 min. Eluted proteins were subjected to electrophoresis in 10% SDS-PAGE and transferred onto nitrocellulose membranes. Membranes were probed overnight at 4 °C with anti-HA antibody (ab9110, abcam, Cambridge, UK) in milk-containing incubation buffer. Membranes were washed three times with PBST, incubated with HRP-conjugated anti-rabbit antibody and milk buffer for 1 h, and washed three more times with PBST for 10 min. Resulting bands were visualized using an enhanced chemiluminescence Western blot detection kit. In order to test for biological activity of natural MCT8-aAb, positive and control serum samples (300 µL each) were diluted with 300 µL of PBS and incubated overnight under constant agitation at 4 °C with protein A sepharose slurry (300 µL per reaction). Formed complexes were pelleted and washed six times with PBS. Bound immunoglobulins were eluted with 25 mM citric acid (pH 3.0) and pH was immediately adjusted to 7.0 using HEPES-NaOH (1M, pH 8.0). Eluted immunoglobulins were concentrated to 300 µL (final protein concentration; 10 mg/mL) using Centricon Filters at 4 °C. Interfering effects of isolated immunoglobulins on TH uptake were tested with MDCK-1 cells expressing recombinant MCT8-HA. To this end, cells were incubated with ^125^I-T4 (PerkinElmer Life LAS GmbH, Rodgau, Germany) and analyzed after different time periods, essentially as described [28].

### 2.6. Statistical Analysis

Statistical analysis was performed using GraphPad Prism v4.0 (GraphPad Software Inc., San Diego, CA, USA) and SAS version 9.4 (SAS Institute, Cary, NC, USA). Data are presented as mean ± SD or median with interquartile range (IQR) as indicated. Normal distribution was tested according to Shapiro–Wilk, and unpaired t-test was used to compare quantitative variables when normal distribution was given. Otherwise, data were compared by two-sided non-parametric U Mann–Whitney Test. Statistical significance is assigned as * *p* < 0.05, ** *p* < 0.01 or *** *p* < 0.001.

## 3. Results

### 3.1. Test for Linearity of the MCT8-aAb and MCT10-aAb Assays with Commercial Antibodies

**Figure 1:**
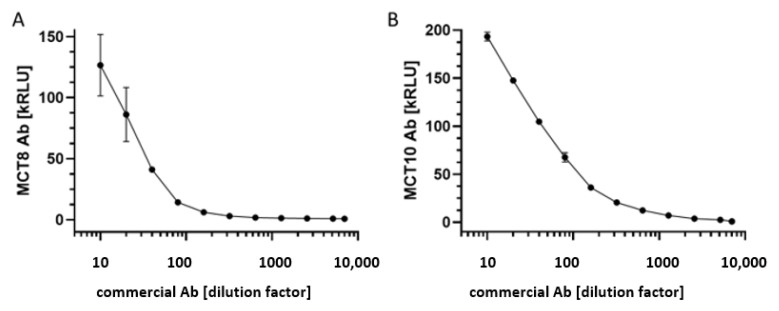
Characterization of the novel assays by a commercial anti-MCT8 or anti-MCT10 antiserum. (**A**) Dilution of the commercial MCT8-Ab with human serum yielded dose-dependent signals, indicating a suitable assay design and acceptable measuring range for clinical samples. (**B**) The commercial antiserum to human MCT10 was similarly suitable for testing the newly generated assay for detecting and quantifying MCT10-aAb in clinical samples. Measurements were conducted in duplicates, kRLU; 1000 relative light units.

One commercially available MCT8-specific and one MCT10-specific antiserum were selected to test the newly generated analytical assays for signal linearity. The signals obtained from the commercial antiserum samples in dilution experiments with human serum as matrix were correlating positively to the antibodies in the novel autoantibody tests with the MCT8-Luc-fusion protein (Figure 1A) and MCT10-Luc-fusion protein (Figure 1B), respectively. The linear range for analysis extended over at least one order of magnitude each, i.e., with an acceptable dynamics for the intended clinical analyses.

### 3.2. Prevalence of aAb to MCT8 and MCT10 in Healthy Subjects

**Figure 2:**
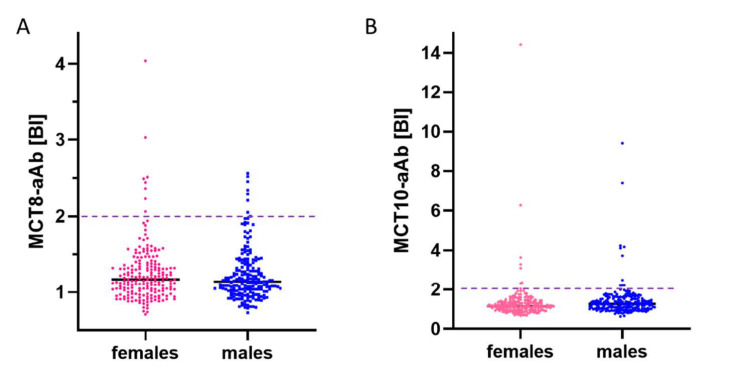
Prevalence of MCT8-aAb and MCT10-aAb in healthy control subjects. A cohort of healthy male and female subjects (*n* = 200 each) were tested in parallel for (**A**) MCT8-aAb, and (**B**) MCT10-aAb. Using the outlier criterion of the 75th percentile (P75) plus 1.5-times the inter quartile range (P75 + 1.5 × IQR), a prevalence of 3.8% for MCT8-aAb, and 4.5% for MCT10-aAb was observed. No sex-specific difference was noted. The binding index (y-axis, factor above background) and the thresholds for positivity (P75 + 1.5 × IQR of all signals, broken horizontal lines) are indicated.

Serum samples from a cross-sectional collection of healthy human subjects (*n* = 400, 50% females) were analyzed by the MCT8-aAb and MCT10-aAb assays in parallel. The signals obtained showed a skewed distribution with some exceptionally high signals, indicating the presence of reactive aAb. Few slightly positive serum samples were identified by the MCT8-aAb assay (Figure 2A), and several strongly positive samples were found by the MCT10-aAb assay (Figure 2B). Using the outlier criterion of the 75th percentile plus 1.5-times the interquartile range (P75 + 1.5 × IQR), a binding index of 2.01 defines the threshold for positivity. Applying this criterion, the prevalence in healthy subjects for MCT8-aAb was 3.8% (15 out of 400; 8 females and 7 males), and 4.5% for MCT10-aAb (18 out of 400; 8 females and 10 males), respectively, with the binding indices of positive signals for MCT10-aAb exceeding those for MCT8-aAb strongly (Figure 2A,B). 

### 3.3. Prevalence of MCT8-aAb and MCT10-aAb in Overweight Young Subjects

**Figure 3:**
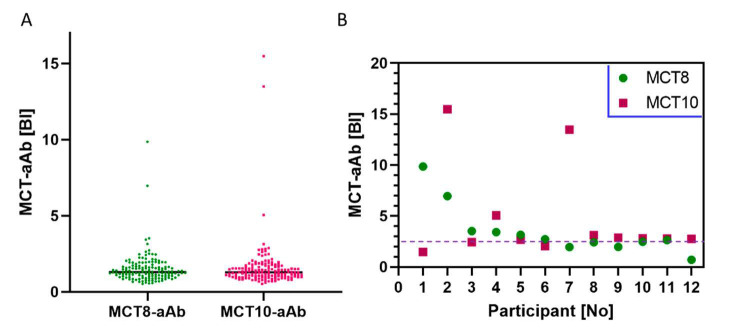
Prevalence of MCT8-aAb and MCT10-aAb in overweight adolescents. (**A**) A cohort of healthy adolescents (*n* = 143) participating in a weight reduction and maintenance program were analyzed in parallel for MCT8-aAb and MCT10-aAb. Using the outlier criterion of P75 plus 1.5-times the inter quartile range (P75 + 1.5 × IQR), a prevalence of 4.2% for MCT8-aAb and 6.3% for MCT10-aAb, respectively, was observed. (**B**) Overview on the individuals being positive for MCT8-aAb or MCT10-aAb, or both. The data indicate that some subjects expressed aAb recognizing both transporter proteins, likely due to the relatively high structural and sequence similarity of the antigens. The binding index (BI) is indicated along with the threshold for positivity (broken horizontal line). Green symbols; MCT8-aAb, red symbols; MCT10-aAb.

Next, a cohort of healthy overweight adolescents (*n* = 143) participating in a weight reduction program (“MAINTAIN”) were analyzed. The signals obtained for both the MCT8-aAb and the MCT10-aAb were not normally distributed and showed again a skewed pattern (Figure 3A). A small number of samples was above the P75 + 1.5 × IQR threshold for MCT8-aAb and MCT10-aAb positivity, i.e., at a binding index greater than 2.66 (BI > 2.66). The number of positive signals for aAb to the two transporters was comparable, yielding a prevalence of 4.2% (6 out of 143) for MCT8-aAb, and 6.3% (9 out of 143) for MCT10-aAb, respectively. Three of the samples were positive for both MCT8-aAb and MCT10-aAb (Figure 3B). The two samples with the highest signals for MCT8-aAb (P1, P2) were selected for further testing on a potential functional role in vitro.

### 3.4. In Vitro Activity of MCT8-aAb Affecting TH Uptake into Cells In Vitro

**Figure 4:**
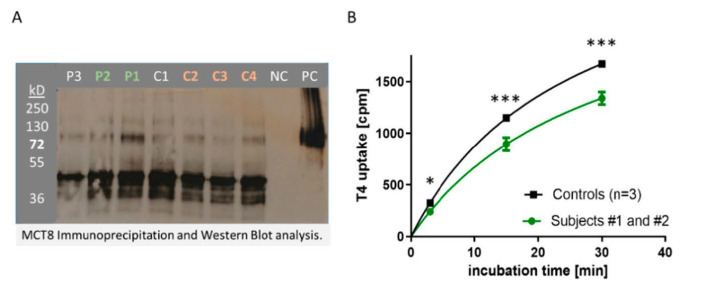
In vitro activity of MCT8-aAb. (**A**) Immunoprecipitation and Western blot analysis of recombinant human MCT8 by serum samples positive (P1–P3) or not (controls; C1–C4) for MCT8-aAb. The thick dark band in the lane far right presents the positive control (PC), i.e., an aliquot of recombinant MCT8 antigen as size marker and loading control. (**B**) Uptake of labeled T4 into MCT8-expressing MDCK-cells in culture in the presence of control (black squares, *n* = 3) or MCT8-aAb positive serum (green dots, P1 and P2). The T4 uptake dynamics were similar, but the extent of uptake was diminished by the presence of immunoglobulins isolated from the MCT8-aAb positive sera. Comparison by two-sided non-parametric U Mann–Whitney Test; * *p* < 0.05, *** *p* < 0.001.

Three positive samples for MCT8-aAb from the cohort of overweight adolescents (P1–P3) along with four negative serum samples (C1–C4) were selected and tested for their interaction with recombinant MCT8 in vitro (Figure 4A). Two of the positive samples (P1, P2) along with three control samples were tested for their potential biological effects on TH uptake in vitro (Figure 4A). To this end, MDCK-cells expressing or not recombinant human HA-tagged MCT8 were grown and harvested. Cell homogenates were incubated with serum containing MCT8-aAb (P1–P3) or control serum (C1–C4). Antibody-protein complexes were precipitated with protein A, protein was eluted with glycine, applied to SDS-PAGE and blotted onto a nitrocellulose membrane. Recombinant MCT8 protein was detected via the fused HA-tag by HA-specific antibody (Figure 4A). 

In order to test for interference with T4 uptake, MDCK-cells expressing recombinant MCT8 were plated and grown to confluency, MCT8-aAb positive or negative serum was added along with radioactively labeled TH (125I-T4), and cells were harvested after different periods of time. The signals obtained (counts per minute, cpm) from the homogenates correspond to the degree of TH uptake and were significantly higher in the cells incubated with MCT8-aAb negative serum (controls, *n* = 3) as compared to cells incubated with MCT8-aAb positive (P1, P2) serum samples (Figure 4B). 

### 3.5. Prevalence of MCT8-aAb and MCT10-aAb in Thyroid Patients

**Figure 5:**
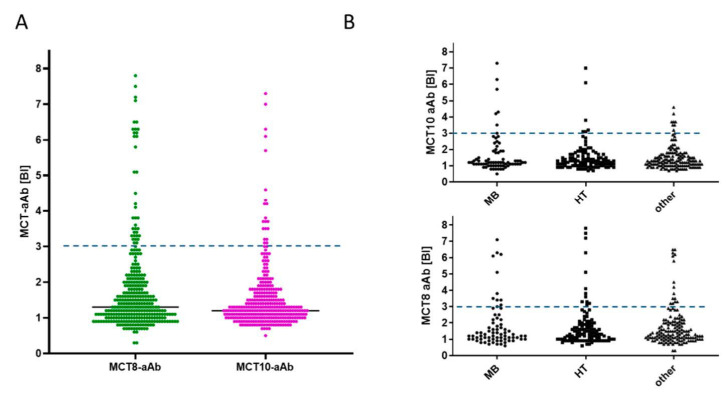
Prevalence of MCT8-aAb and MCT10-aAb in thyroid patients. (**A**) A cohort of adult patients with different thyroid diseases (*n* = 318) were analyzed for the presence of MCT8-aAb and MCT10-aAb, respectively. Several highly positive serum samples were identified, surpassing the diagnostic threshold. (**B**) Separating the patients according to their primary diagnosis into Graves’ disease (MB), Hashimoto’s thyroiditis (HT) and other thyroid diseases (other) indicated positive samples in all disease groups with no apparent disease-specific prevalence for MCT8-aAb or MCT10-aAb. The binding index (BI) is indicated along with the threshold for positivity (broken horizontal line), i.e., the value representing P75 + 1.5 × IQR of the full study cohort.

Finally, the prevalence of MCT8-aAb and MCT10-aAb was determined in a cohort of adult thyroid patients. The signal distribution was similarly skewed as observed with the other two cohorts (Figure 5). Positive MCT8-aAb were found with a prevalence of 11.9% (38 out of 318), and MCT10-aAb with a prevalence of 6.3% (20 out of 317) in serum of thyroid patients (Figure 5A). Subdividing the full study group into different thyroid diseases, the prevalence of samples highly positive for MCT8-aAb was similar in the groups of Graves’ disease (*n* = 11), Hashimoto’s thyroiditis (*n* = 12) and other thyroid patients (*n* = 15) (Figure 5B). Similarly, there was no obvious difference in relation to MCT10-aAb, and patients with Graves’ disease (*n* = 6), Hashimoto’s thyroiditis (*n* = 6) or other thyroid disease (*n* = 8) showed a similar prevalence of MCT10-aAb (Figure 5B).

## 4. Discussion

In this explorative study, we present evidence that MCT8 and MCT10 are recognized as autoantigens in a subset of human subjects, and that MCT8-aAb and MCT10-aAb are similarly prevalent in obese adolescents and healthy adults. As we did not include a control group of non-obese adolescents, the influence of age and obesity on MCT8-aAb and MCT10-aAb prevalence remains unresolved. In thyroid patients, prevalence for both MCT8-aAb and MCT10-aAb was two- to three-fold higher than in healthy controls or adolescents, but still relatively low as compared to established thyroid autoantibodies. However, in this first analysis, no specific difference in relation to thyroid disease diagnosis was observed and no significant relation to any of the measured TH axis biomarkers (total T4, TSH) was detected. Both assays have been partially characterized with a specific commercial antiserum each, enabling an independent replication and comparison of these first results by other research teams interested in this issue. The in vitro experiment indicated that natural MCT8-aAb are capable of interfering with T4 uptake, suggesting a potential contribution of MCT8-aAb to the biological TH status and parameters of feedback regulation. However, the aAb detected in this study were of moderate concentrations only, and no obvious laboratory or clinical phenotype was associated with aAb positivity. As the nature of this pilot study was a cross-sectional setting with three small cohorts of samples, a potential role for disease predisposition or disease course cannot be deduced yet, and longitudinal analyses of larger cohorts of samples are needed next to identify a potential pathogenic relevance of MCT8-aAb or MCT10-aAb or both.

Nevertheless, the identification of specific MCT8-aAb and MCT10-aAb highlights that the TH transporters can be classified as novel autoantigens, thereby introducing another natural factor potentially affecting TH release and signaling. It is well established that individuals differ profoundly in their set-points of the TH axis [29], and their response to TH treatment [30]. Reference ranges are useful for detecting strong deviations of single subjects as compared to the majority in a given population, but do not faithfully replicate the individual TH status. Individual variations in e.g., serum TSH are in a more restricted concentration range as the boundaries of normal reference ranges would suggest. Molecular reasons for the differences in individual set-points of the TH axis are commonly explained by different genotypes and modifying effects of certain single nucleotide polymorphisms (SNPs) in the centrally involved genes [31,32]. Accordingly, SNPs in the genes encoding the TRH-receptor (TRHR) [33], deiodinase type 1 or 2 [34,35], the TH transport molecules [10,36] or enzymes involved in TH metabolism have been described to affect peripheral TH status and individual set points [37]. Apart from stable SNPs and specific genotypes in the populations, a few inherited gene defects have highlighted the importance of particular genes in TH feedback control, causing variable degrees of resistance-to-TH (RTH), including the TH receptors alpha [38] or beta [39], the MCT8 [40] or an RNA-binding protein involved in deiodinase biosynthesis [41]. Besides these inborn differences in the genotypes of central components of the TH axis, epigenetic effects have likewise been put forward for explaining some variability in the TH axis, e.g., in relation to TRH [42], the TSH-receptor [43], TH receptor beta [44] or deiodinase type 3 [45]. In this manuscript, we highlight the notion that MCT8-aAb or MCT10-aAb may add another component to the observed variability of TH feedback regulation in a given population. A considerable fraction of healthy and especially of diseased subjects are positive for aAb to the most prominent TH transporters known to date, i.e., to MCT-8 and MCT-10, respectively, or to both in parallel. The aAb titers and specific binding characteristics constitute plausible parameters affecting TH feedback regulation and the individual TH set-points.

The identification of MCT8-aAb and MCT10-ab is not very surprising, as there is an increasing number of well-characterized examples of clinically relevant aAb to endocrine-relevant membrane proteins. Besides the already mentioned TSH-receptor, also the thyroid transporters for iodide, i.e., the sodium-iodide symporter (NIS) and pendrin, have been described as potential autoantigens of the TH axis, and reliable test systems have been developed [26,46]. Initially, strongly varying and particularly high prevalence has been reported in thyroid patients, partly due to the usage of small peptides as bait molecules in the aAb assays. Subsequent studies using full-length proteins either by radioactive ligand binding [46] or via detection of antigen-reporter fusion proteins [26] indicated a similar and moderate prevalence, and in a range compatible to what is reported in this manuscript for MCT8-aAb and MCT10-aAb, respectively. Longitudinal studies for elucidating a potential role of such aAb for thyroid disease incidence are consistently lacking, but it is not unlikely that transporter-targeting aAb to iodide or TH uptake are eliciting biological activity in human subjects. The aAb may act alone or in combination and thereby contribute to the set-point of TH status and feedback regulation in a personalized way, thereby affecting the individual endocrine status of T3, T4 and TSH [47].

This notion is compatible to other membrane bound transport autoantigens known to be relevant for endocrine disease risk assessment and prediction, e.g., the zinc transporter ZnT8 in autoimmune diabetes mellitus [48,49]. Positive autoimmunity against ZnT8 is associated with an increased disease risk and high insulin requirement [50], and detection of ZnT8-aAb in siblings from affected children indicates an unfavorable prognosis [51]. Similarly, aAb recognizing transporters of the low-density-lipoprotein-receptor-related protein family (LPR) have been implicated in systemic autoimmune diseases, e.g., in rheumatoid arthritis [52]. Specific stimulating aAb to adrenergic receptors (AdrR) have been identified in Chagas’ disease, i.e., in a most prevalent endemic autoimmune disease of the Americas [53]. However, detection of AdrR-aAb is difficult, and routine tests for in vitro analysis are not yet available [54]. Convincing evidence has been obtained from bioassays, where the spontaneous rhythmic beating of cultured neonatal rodent cardiomyocytes is monitored and related to stimulating AdrR-aAb [55]. Conversely, inhibiting AdrR-aAb have been postulated in chronic fatigue syndrome, where they may impair adrenergic signaling [56], but a validation of the proposed diagnostic tests is missing. 

Another prominent example for signal relevant aAb to a membrane ion channel is Myasthenia gravis, where aAb to the acetylcholine receptor are interfering with signal transduction at the neuromuscular junction [57]. Strong disease activity may suddenly occur, e.g., upon infection, potentially leading to an acute neurological emergency of respiratory failure and myasthenic crisis [58]. Fortunately, therapeutic measures have been improved, and disease severity can successfully be controlled by pharmacological intervention [59]. We have recently studied aAb to the IGF1-receptor (IGF1R), impairing IGF1 signaling [25], and related the presence of IGF1R-aAb to poor physical strength in young subjects [27]. Additional physiological effects are likely, given the broad relevance of IGF1 in development and disease. This notion is supported by the characterization of type B insulin resistance, where a high aAb load to the insulin receptor may constitute a life-threatening condition [60]. Fortunately, current therapeutic interventions are capable of controlling an overwhelming aAb burden with obviously long-term health benefits [61]. These findings are of general relevance to the TH axis, once subjects with exceedingly high levels and endocrine disrupting activities of MCT8-aAb or MCT10-aAb are identified. The novel assays for MCT8-aAb and MCT10-aAb will facilitate the identification and therapeutic control of such subjects with interfering natural autoimmunity to TH uptake.

## 5. Conclusions

We conclude that a subset of obese adolescents and adults express detectable amounts of MCT8-aAb or MCT10-aAb or both, which may affect the individual set point of the TH axis. The increased prevalence of MCT8-aAb and MCT10-aAb observed in thyroid patients points to a potential diagnostic relevance of the aAb, even though their presence was not associated with a particular form of thyroid disease, and very high concentrations were not observed in the small clinical cohorts analyzed. Nevertheless, such aAb may be present in certain subjects with unexplainable resistance to TH. The novel assays generated are capable of high throughput screening analyses, are highly robust and sensitive, and form a solid basis for expanding the analyses to large prospective epidemiological studies. These need to be conducted next, in parallel to more focused research on subjects with rare patterns of TH status and TH-related phenotypes that may be caused by a non-genetic but autoimmune-related inhibition of TH transport. 

## Data Availability

The data presented in this study are available on request from the corresponding author. The data are not publicly available due to data safety reasons.
